# Nitrative DNA damage in lung epithelial cells exposed to indium nanoparticles and indium ions

**DOI:** 10.1038/s41598-020-67488-3

**Published:** 2020-07-01

**Authors:** Sharif Ahmed, Hatasu Kobayashi, Tahmina Afroz, Ning Ma, Shinji Oikawa, Shosuke Kawanishi, Mariko Murata, Yusuke Hiraku

**Affiliations:** 1 Department of Environmental and Molecular Medicine, Mie University Graduate School of Medicine, Tsu, Mie Japan; 2 Department of Pharmacy, Daffodil International University, Dhaka, Bangladesh; 3 Faculty of Nursing, Suzuka University of Medical Science, Suzuka, Mie Japan; 4 Faculty of Pharmaceutical Sciences, Suzuka University of Medical Science, Suzuka, Mie Japan; 5 Department of Environmental Health, University of Fukui School of Medical Sciences, Eiheiji, Fukui Japan

**Keywords:** Cancer, Molecular biology, Environmental sciences

## Abstract

Indium compounds have been widely used in manufacturing displays of mobile phones, computers and televisions. However, inhalation exposure to indium compounds causes interstitial pneumonia in exposed workers and lung cancer in experimental animals. 8-Nitroguanine (8-nitroG) is a mutagenic DNA lesion formed under inflammatory conditions and may participate in indium-induced carcinogenesis. In this study, we examined 8-nitroG formation in A549 cultured human lung epithelial cells treated with indium compounds, including nanoparticles of indium oxide (In_2_O_3_) and indium-tin oxide (ITO), and indium chloride (InCl_3_). We performed fluorescent immunocytochemistry to examine 8-nitroG formation in indium-exposed A549 cells. All indium compounds significantly increased 8-nitroG formation in A549 cells at 5 ng/ml after 4 h incubation. 8-NitroG formation was largely reduced by 1400 W, methyl-β-cyclodextrin (MBCD) and monodansylcadaverine (MDC), suggesting the involvement of nitric oxide synthase and endocytosis. 8-NitroG formation in A549 cells was also largely suppressed by small interfering RNA (siRNA) for high-mobility group box-1 (*HMGB1*), receptor for advanced glycation and end products (*AGER*, RAGE) and Toll-like receptor 9 (*TLR9*). These results suggest that indium compounds induce inflammation-mediated DNA damage in lung epithelial cells via the HMGB1-RAGE-TLR9 pathway. This mechanism may contribute to indium-induced genotoxicity in the respiratory system.

## Introduction

In the last few decades, indium compounds created the demand for extensive usage in a variety of products, including liquid crystal displays of mobile phones, computers and televisions^1^. Indium compounds have been used in the form of indium-tin oxide (ITO), a sintered material, consisting approximately 90% of indium oxide (In_2_O_3_) and 10% of tin oxide (SnO_2_), due to its characteristics of high electrical conductivity, transparency and mechanical resistance^2^. According to the statistics of U.S. Geological Survey (USGS), world production of indium has increased for recent years^3^, and world refinery production of indium is 750 tons in 2018^4^. ITO accounted for 90% of the total indium demand in Japan^5^. Although the production of indium compounds is expected to rise significantly because of increasing use in electronic devices, health hazard associated with occupational exposure to these compounds is a great concern. In 2003, the first case of indium-related interstitial pneumonia caused by occupational inhalation exposure to ITO was reported in Japan^6^. An epidemiological study was conducted at an indium-processing factory, and revealed dose-dependent emphysematous change due to indium exposure^7^. Clinical cases of lung disease associated with indium workers have been reported in Japan, China and United States^8–11^.

Carcinogenicity of indium compounds has been demonstrated in animal experiments. A long-term inhalation of indium phosphide caused lung cancer in animals, and this compound has been classified as a group 2A carcinogen (probably carcinogenic to humans) by the International Agency for Research on Cancer (IARC)^12^. ITO caused lung carcinoma in rats after inhalation exposure to for 2 years^13^. Recently, IARC has classified ITO as a group 2B carcinogen (possibly carcinogenic to humans)^14^. These findings raise a concern that indium compounds exhibit lung carcinogenicity in humans.

Accumulation of indium compounds in the lung tissue causes chronic inflammation^5^. Chronic inflammation is known to contribute to a substantial part of cancer cases worldwide^15^. In inflammation-related carcinogenesis, reactive oxygen species (ROS) and reactive nitrogen species (RNS) generated from inflammatory and epithelial cells play a critical role. These reactive species form mutagenic DNA lesions, including 8-nitroguanine (8-nitroG), via the interaction with DNA bases^16,17^. 8-NitroG is formed by the interaction of guanine with peroxynitrite (ONOO^−^), which is generated by the reaction of nitric oxide (NO) and superoxide (O_2_^**·**−^)^18^. We have reported that 8-nitroG is formed at the sites of carcinogenesis in a wide variety of animal models and clinical specimens of cancer-prone inflammatory diseases and proposed that this DNA lesion can be a potential biomarker of inflammation-related cancer^19,20^. The objective of this study was to determine the genotoxic effects of indium compounds on lung epithelial cells, and clarify the molecular mechanism. We have recently reported that In_2_O_3_ induced 8-nitroG formation in mouse macrophages^21^. In this study, we used In_2_O_3_ and ITO nanoparticles as representative indium compounds to examine 8-nitroG formation in human lung epithelial cells. Tabei et al. have demonstrated that intracellular accumulation of indium ions, which are released from indium-containing particles, contributes to DNA damage^22^. Therefore, we also used indium chloride (InCl_3_) to examine the ability of ionic indium to cause nitrative DNA damage in this study.

High mobility group box-1 (HMGB1) is a nuclear protein, which is released from damaged or necrotic cells and associated with inflammatory diseases and cancer^23^. HMGB1 forms a complex with DNA, and the HMGB1-DNA complex binds to receptor for advanced glycation end products (RAGE), which is a multi-ligand receptor on cell membrane involved in cancer, sepsis and other diseases^24^. Toll-like receptor 9 (TLR9) is located on the lysosomal membrane and involved in cancer, sepsis and other diseases. This receptor mediates inflammatory responses against a wide variety of infectious and non-infectious agents via interaction with CpG DNA of exogenous and endogenous origin^24–26^. Our recent study has demonstrated that the HMGB1-RAGE-TLR9 signaling pathway was involved in nitrative DNA damage in human lung epithelial cells treated with multi-walled carbon nanotube (MWCNT)^27^. To clarify whether this pathway is involved in indium-induced DNA damage, we examined inhibitory effects of small interfering RNA (siRNA) for these molecules on 8-nitroG formation.

## Results

### Dispersion of indium nanoparticles and size distribution

**Figure 1:**
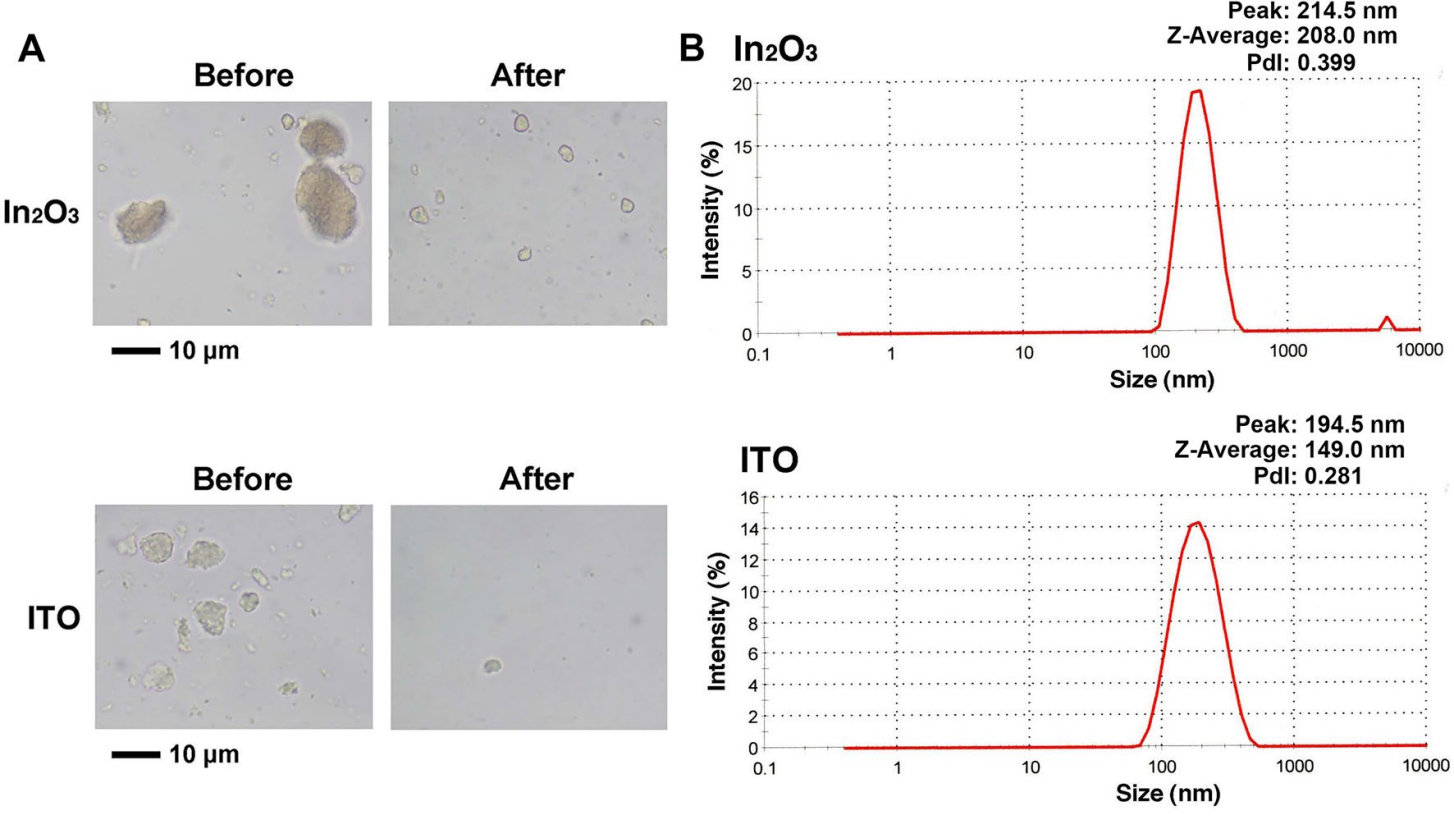
Dispersion of In_2_O_3_ and ITO particles and their size distribution. (**A**) In_2_O_3_ and ITO agglomerates before and after the sonication. In_2_O_3_ and ITO suspensions in DMEM containing heat-inactivated FBS and kanamycin were vortexed (before) and sonicated (after) as described in “Methods” section. In_2_O_3_ and ITO agglomerates were observed with a light microscope. Bar = 10 µm. (**B**) Size distribution of dispersed In_2_O_3_ and ITO particles. The size distribution was measured with a Zetasizer Nano particle size analyzer (Malvern Worcestershire UK).

In_2_O_3_ and ITO nanoparticles were suspended in Dulbecco’s Modified Eagles Medium (DMEM) containing fetal bovine serum (FBS) and kanamycin, and agglomerates were dispersed with an ultrasonic homogenizer. Figure 1A shows In_2_O_3_ and ITO agglomerates before and after sonication. After sonication, the particles were dispersed into submicron-sized particles, capable of reaching human alveolus. Figure 1B shows size distribution of dispersed In_2_O_3_ and ITO agglomerates analyzed with a particle size analyzer. The values concerning size distribution [peak, Z-average and polydispersity indexes (PdI)] of these compounds were as follows: In_2_O_3_ (peak, 214.5 nm; Z-average, 208.0 nm; PdI, 0.399); ITO (peak, 194.5 nm; Z-average, 149.0 nm; PdI, 0.281).

### Cytotoxic effect of indium compounds

Cytotoxic effects of indium compounds on A549 human lung epithelial cells were evaluated by 3-(4,5-dimethylthiazol-2-yl)-2,5-diphenyltetrazolium bromide (MTT) assay. A549 cells were treated with 5–50 µg/ml of indium compounds (In_2_O_3_, ITO and InCl_3_) for 24 h. These compounds did not significantly decrease cell viability and there was no significant difference in the viability among these compounds under the conditions used (two-way ANOVA, Supplementary Figure S1 online).

### 8-NitroG formation in indium-treated cells

**Figure 2:**
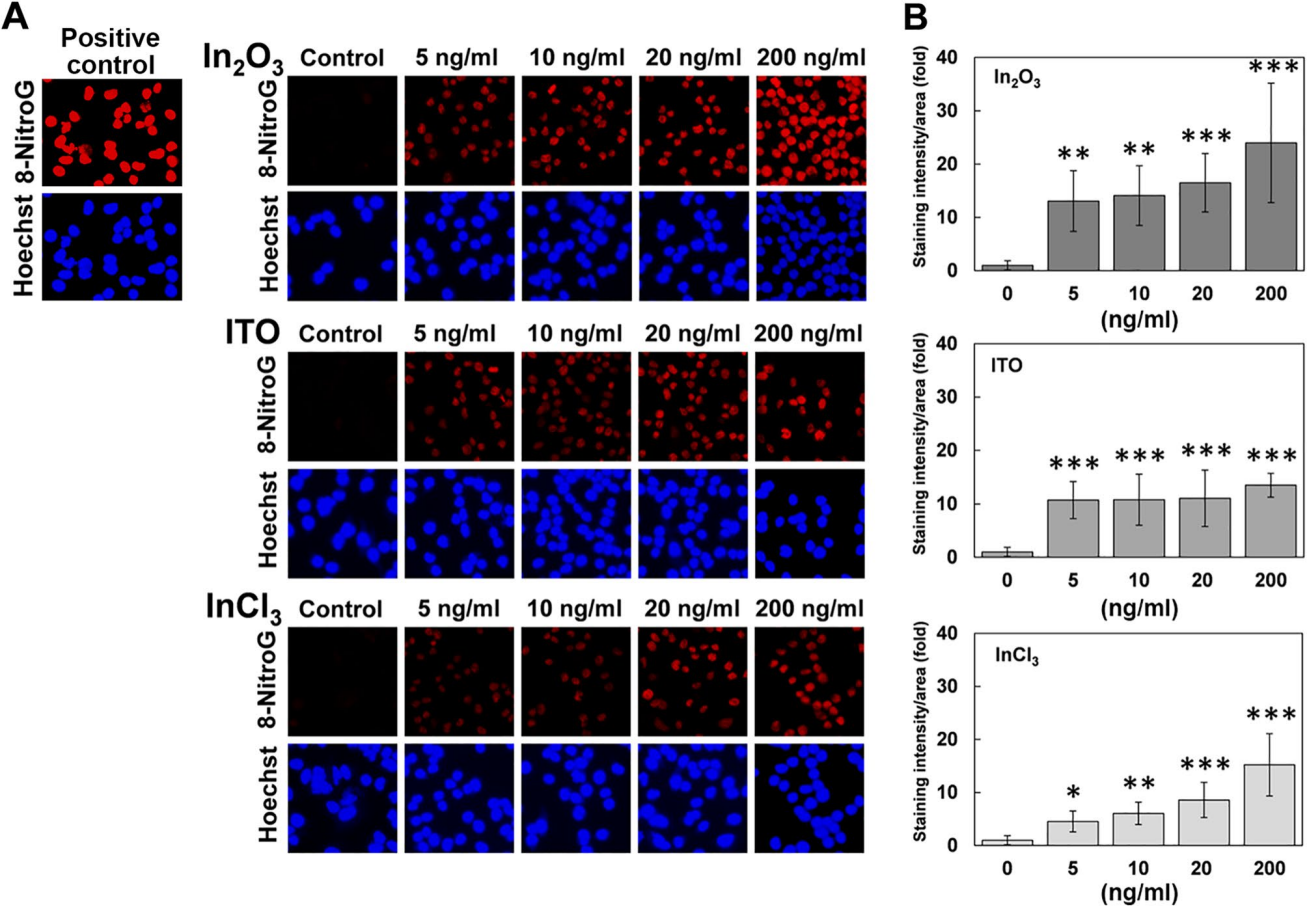
8-NitroG formation in indium-treated cells. A549 cells were incubated with the indicated concentrations of In_2_O_3_, ITO and InCl_3_ for 4 h at 37 °C. Positive control was prepared by incubating A549 cells with culture supernatant of MNCNT-exposed cells as described in “Methods” section. 8-NitroG formation was detected by immunocytochemistry as described in “Methods” section. (**A**) Fluorescent images of indium-induced 8-nitroG formation in A549 cells. The red fluorescence shows 8-nitroG formation and the blue fluorescence shows the nucleus stained with Hoechst 33258. Magnification, × 200. (**B**) Quantitative image analysis for indium-induced 8-nitroG formation in A549 cells. The staining intensity per area was quantified with an ImageJ software, and the relative intensity of the control was set at 1. The data were expressed as means ± SD of 4–8 independent experiments. *p < 0.05, **p < 0.01 and ***p < 0.001 compared with the control by ANOVA followed by Tukey’s test.

We performed immunocytochemical analysis for 8-nitroG formation in A549 cells treated with indium compounds. Figure 2A shows fluorescent images of 8-nitroG formation in indium-treated cells. Positive control shows 8-nitroG formation in A549 cells incubated in culture supernatant of MWCNT-exposed cells, which was prepared as reported previously^27^. Clear fluorescence was observed at 5 ng/ml (equivalent to 1.42 ng/cm^2^) in In_2_O_3_-, ITO- and InCl_3_-treated cells. No or weak staining was observed in non-treated control. The staining pattern of 8-nitroG overlapped with that of Hoechst 33258, suggesting that 8-nitroG was formed mainly in the nucleus (Fig. 2A). Image analysis revealed that the staining intensity of 8-nitroG in In_2_O_3_-, ITO- and InCl_3_-treated A549 cells was significantly increased at 5 ng/ml compared with the non-treated control (*p* < 0.05, Fig. 2B). In_2_O_3_ and InCl_3_ induced 8-nitroG formation in a dose-dependent manner, whereas ITO caused 8-nitroG formation to a similar extent at 5–200 ng/ml.

### Time course of 8-nitroG formation

**Figure 3:**
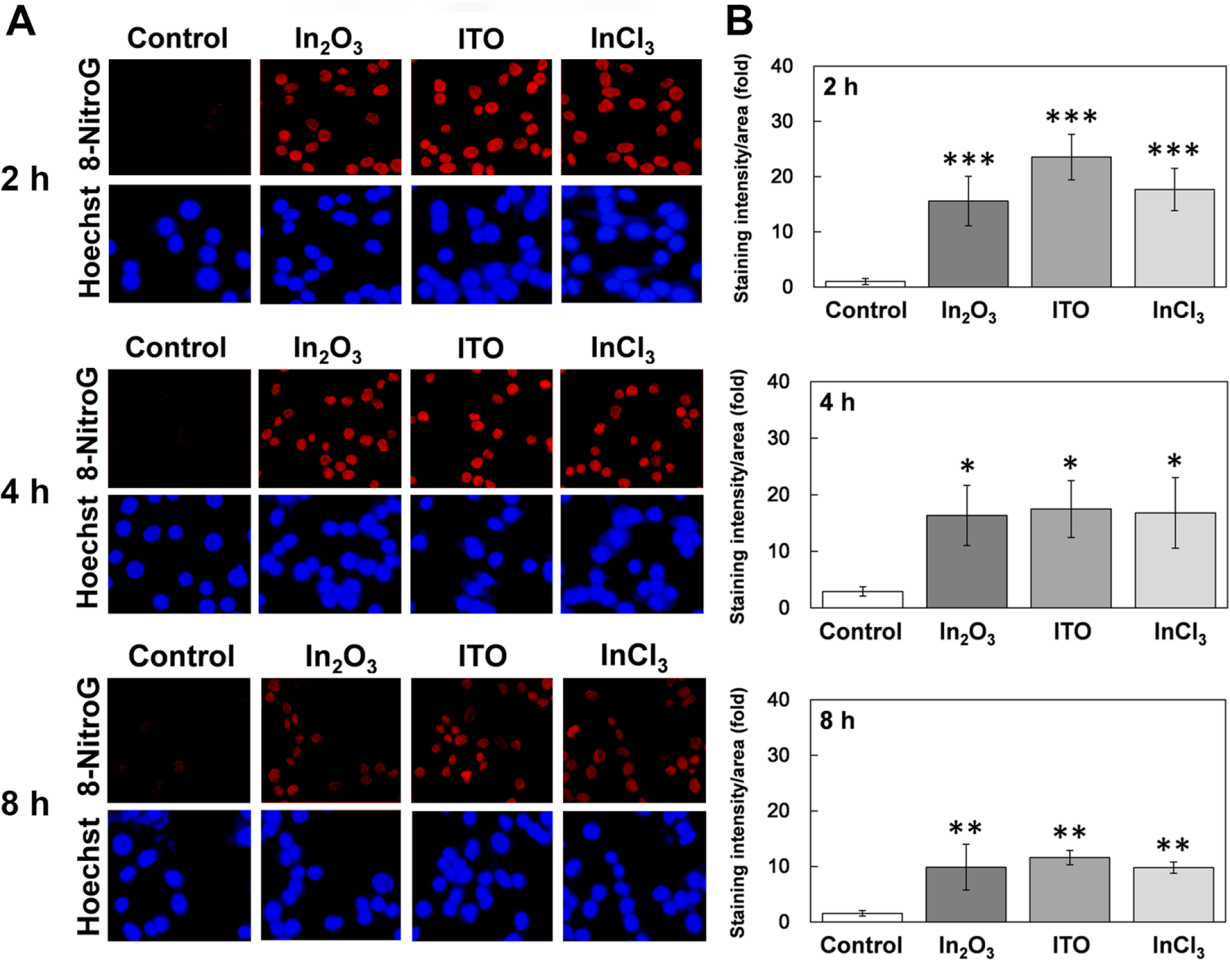
Time course of 8-nitroG formation in indium-treated A549 cells. (**A**) Fluorescent images of indium-treated A549 cells at different incubation times. A549 cells were treated with 200 ng/ml of In_2_O_3_, ITO and InCl_3_ at 37 °C for indicated durations. 8-NitroG was detected by immunocytochemistry as described in “Methods” section. The nucleus was stained with Hoechst 33258. Magnification, × 200. (**B**) Quantitative image analysis of 8-nitroG formation in indium-treated A549 cells. Staining intensities of 8-nitroG per area were analyzed with an ImageJ software. The relative intensity of the control at 2 h was set at 1. The data were expressed as means ± SD of 3–4 independent experiments. *p < 0.05, **p < 0.01 and ***p < 0.001 compared with the control by ANOVA followed by Tukey’s test.

Figure 3A shows fluorescent images of 8-nitroG formation in A549 cells treated with indium compounds for different durations. All indium compounds induced clear 8-nitroG formation at 2, 4 and 8 h. The staining intensity of 8-nitroG formation in indium-treated cells was significantly greater after 2–8 h incubation than that in non-treated control (*p* < 0.05, Fig. 3B). Staining intensity of 8-nitroG tended to be weaker at 8 h than those at 2 and 4 h (Fig. 3B).

### Effects of inducible nitric oxide synthase (iNOS) and endocytosis inhibitors on indium-induced 8-nitroG formation

**Figure 4:**
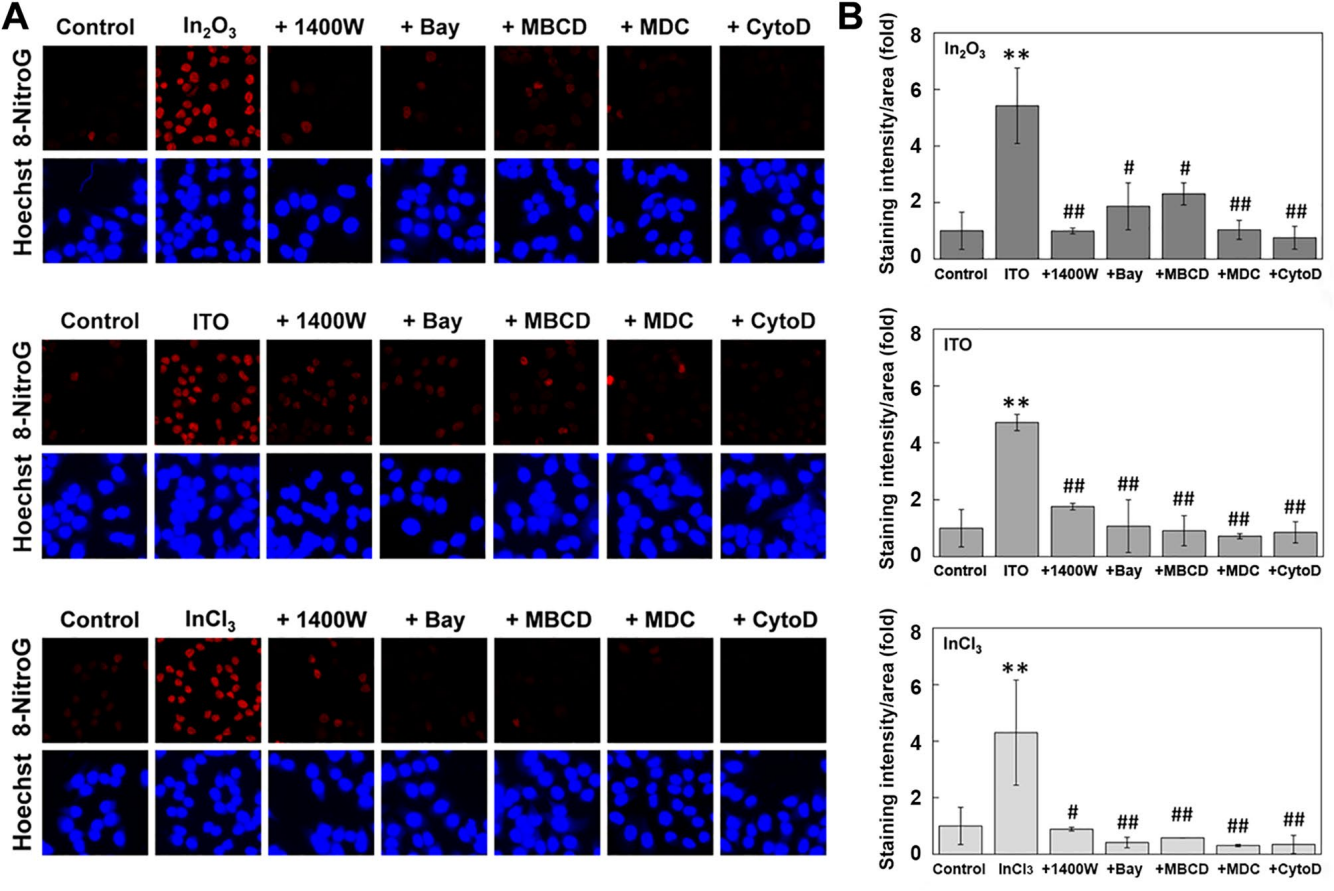
Effects of iNOS and endocytosis inhibitors on indium-induced 8-nitroG formation. (**A**) Fluorescent images of 8-nitroG formation in indium-treated A549 cells. A549 cells were treated with 200 ng/ml of In_2_O_3_, ITO and InCl_3_ for 4 h at 37 °C. The cells were co-treated with 1400 W, Bay, MBCD, MDC and CytoD and 8-nitroG formation was detected by immunocytochemistry as described in “Methods” section. The nucleus was stained with Hoechst 33258. Magnification, × 200. (**B**) Quantitive image analysis for the effects of iNOS and endocytosis inhibitors on indium-exposed A549 cells. The staining intensity per area was quantified with an ImageJ software, and the relative intensity of the control was set at 1. The data were expressed as means ± SD of 3–4 independent experiments. **p < 0.01 versus control and ^#^*p* < 0.05, ^##^*p* < 0.01 versus indium-treated cells by ANOVA followed by Tukey’s test.

To clarify the roles of iNOS expression and endocytosis in indium-induced DNA damage, we examined the effects of inhibitors for these events on 8-nitroG formation in A549 cells. In_2_O_3_, ITO and InCl_3_ induced clear 8-nitroG formation and its immunoreactivity was largely suppressed by the treatment with inhibitors of iNOS (1400 W) and its transcription factor NF-κB (Bay11-7082, Bay), suggesting that iNOS expression was involved in DNA damage. 8-NitroG formation was also suppressed by inhibitors of caveolae-mediated endocytosis (methyl-β-cyclodextrin, MBCD), clathrin-mediated endocytosis (monodansylcadaverine, MDC) and actin polymerization (cytochalasin D, CytoD) (Fig. 4A). Image analysis revealed that these inhibitors significantly reduced indium-induced 8-nitroG formation (*p* < 0.05, Fig. 4B).

### Effects of siRNAs on indium-induced 8-nitroG formation

**Figure 5:**
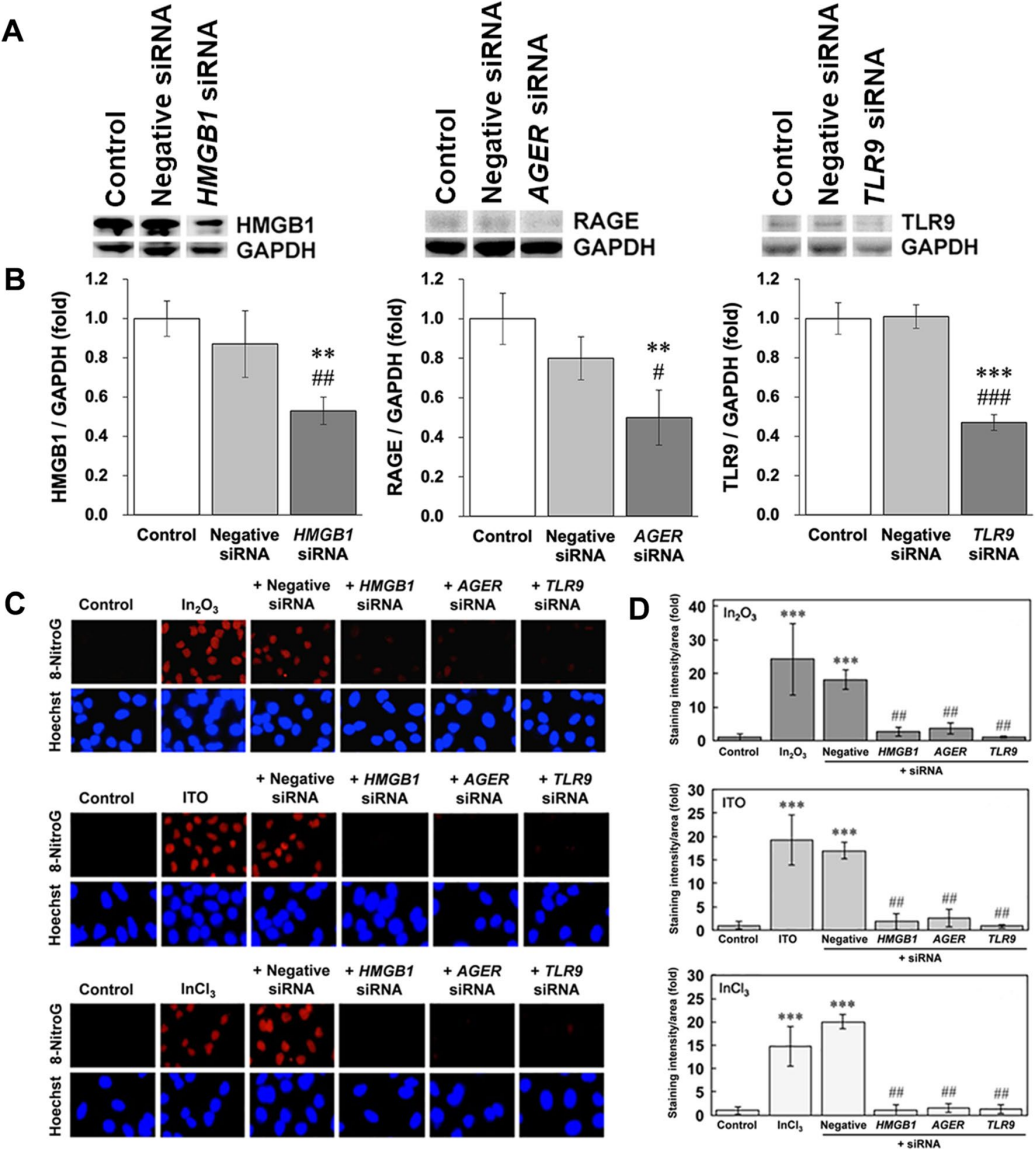
Effects of siRNA on 8-nitroG formation in indium-treated A549 cells. (**A**) Reduction in HMGB1, RAGE and TLR9 expression by siRNA transfection into A549 cells. Effects of siRNA on protein expression were evaluated by Western blotting. These blots were cropped from different parts in the same gel, and each blot was divided with white lines. Full-length blots are shown in Supplementary Figure S2 online. (**B**) Image analysis for HMGB1, RAGE and TLR9 expression in siRNA-transfected A549 cells. These values were expressed as fold changes compared with control. (**C**) Fluorescent images of 8-nitroG formation in indium-treated A549 cells and effects of siRNA. Cells were transfected with 10 nM siRNA for *HMGB1*, *AGER* and *TLR9* or negative control siRNA for 2 days and then treated with 200 ng/ml indium compounds for 4 h as described in “Methods” section. 8-NitroG formation was evaluated by immunocytochemistry as described in “Methods” section. The nucleus was stained with Hoechst 33258. Magnification, × 200. (**D**) Quantitative image analysis for the effects of siRNA on 8-nitroG formation in indium-treated A549 cells. Staining intensities of 8-nitroG per area were analyzed with an image J software. The relative intensity of the control was set at 1. (**B**, **D**) The data were expressed as means ± SD of 3–4 independent experiments. **p < 0.01, ***p < 0.001 versus control and #*p* < 0.05, ^##^*p* < 0.01, ^###^*p* < 0.01 versus negative control siRNA by ANOVA followed by Tukey’s test.

To clarify the mechanism of indium-induced DNA damage, we examined the inhibitory effects of *HMGB1*, *AGER* and *TLR9* siRNA on 8-nitroG formation. Western blotting revealed that transfection of siRNAs for these genes reduced their expression levels, and negative control siRNA had no or weak inhibitory effect (Fig. 5A). Image analysis shows these siRNAs significantly reduced the expression of the corresponding proteins compared with control and negative control siRNA (*p* < 0.05, Fig. 5B). Indium compounds induced clear 8-nitroG formation in A549 cells, and its immunoreactivity was largely reduced by the transfection with *HMGB1*,* AGER* and *TLR9* siRNA (Fig. 5C). Image analysis revealed that negative control siRNA did not affect indium-induced 8-nitroG formation and that transfection of siRNAs for these genes significantly reduced 8-nitroG formation (*p* < 0.01, Fig. 5D).

### Effects of anti-HMGB1 and RAGE antibodies on indium-induced 8-nitroG formation

**Figure 6:**
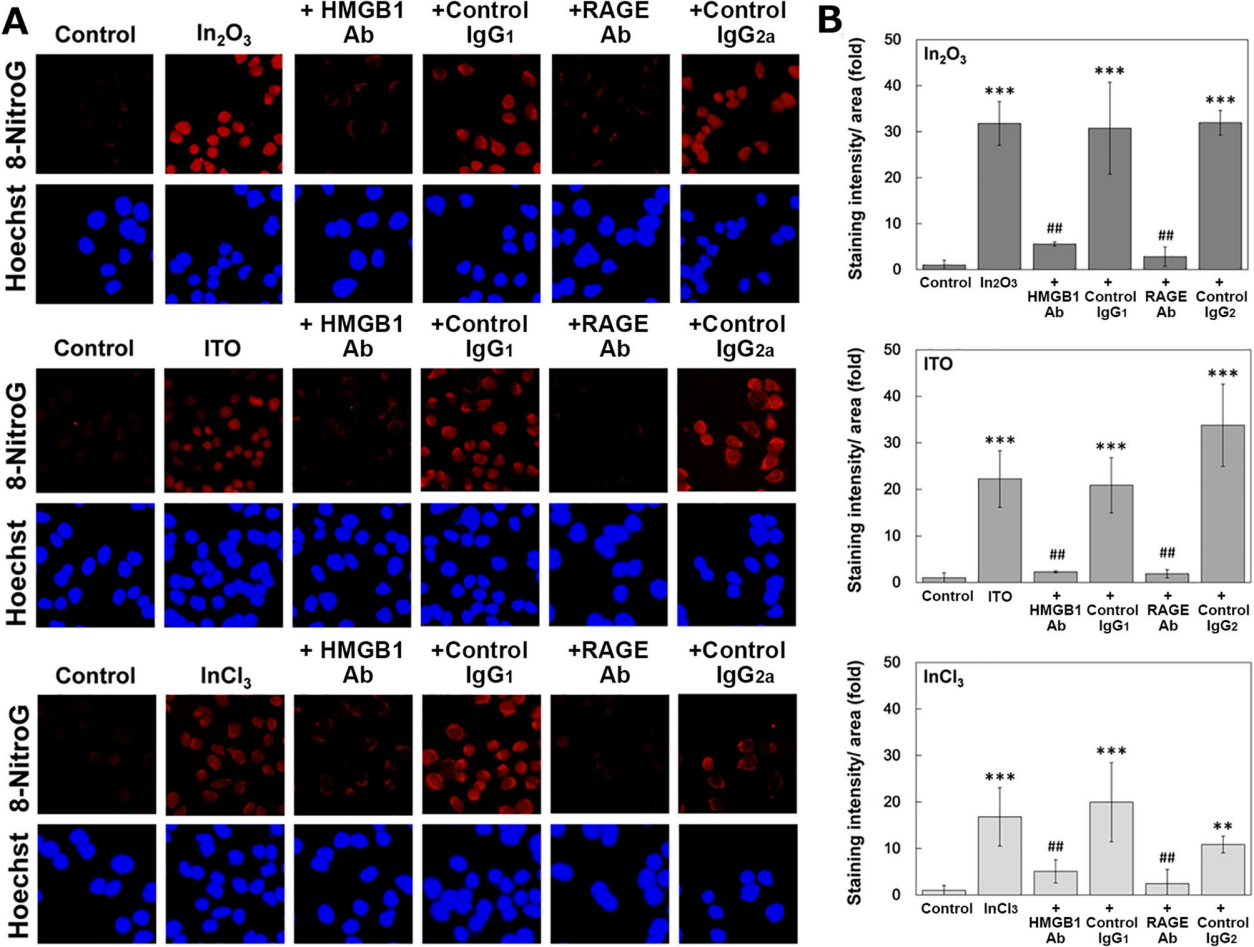
Effects of HMGB1 and RAGE antibodies on 8-nitroG formation in indium-treated A549 cells. (**A**) Fluorescent images of 8-nitroG formation in indium-treated A549 cells and effects of antibodies. A549 cells were pretreated with 10 µg/ml of anti-HMGB1 and anti-RAGE antbodies and their isotype control IgGs for 30 min, followed by the treatment with 200 ng/ml of In_2_O_3_, ITO and InCl_3_ as described in “Methods” section. 8-NitroG was detected by immunocytochemistry. The nucleus was stained with Hoechst 33258. Magnification × 200. (**B**) Quantitative image analysis for the effects of antbodies on 8-nitroG formation in indium-treated A549 cells. Staining intensities of 8-nitroG per area were analyzed with an ImageJ software. The relative intensity of the control was set at 1. The data were expressed as means ± SD of 3–4 independent experiments. **p < 0.01, ***p < 0.001 versus control and ^##^*p* < 0.01 versus negative control by ANOVA followed by Tukey’s test.

To confirm the role of the HMGB1-RAGE-TLR9 pathway in indium-induced DNA damage, we examined the effect of antibodies against HMGB1 and RAGE. In A549 cells, indium compounds induced clear 8-nitroG formation and its immunoreactivity was largely decreased by the pretreatment with antibodies against HMGB1 and RAGE (Fig. 6A). Image analysis shows that isotype control IgGs did not affect indium-induced 8-nitroG formation and antibodies against HMGB1 and RAGE significantly reduced 8-nitroG formation (*p* < 0.01, Fig. 6B).

## Discussion

Indium-containing particles are used extensively in the microelectronic industry. However, interstitial pneumonia and lung cancer occurred after inhalation exposure of workers and experimental animals, respectively. In this study, to clarify the mechanism of indium-induced carcinogenesis, we investigated the genotoxic effects of In_2_O_3_ and ITO nanoparticles and InCl_3_ in A549 human lung epithelial cells. We observed that all indium compounds significantly induced the formation of 8-nitroG in the nucleus of A549 cells. Our group has previously reported that particulate materials, such as MWCNT^27,28^ and carbon black^29^, induced 8-nitroG formation in lung epithelial cells. In this study, we have first demonstrated that not only In_2_O_3_ and ITO particles but also InCl_3_ induced clear 8-nitroG formation in lung epithelial cells. A previous study has shown that cellular uptake and solubilization of indium-containing particles, including ITO, via lysosomal acidification, leading to the release of indium ions, is needed for cytotoxicity^22,30^. Oxidative stress was induced by the accumulation of intracellular indium ions and mediated DNA damage evaluated by Comet assay^22^. These findings suggest that 8-nitroG formation caused by indium compounds can be accounted for by not only their particulate properties but also indium ions, derived from InCl_3_ and released from In_2_O_3_ and ITO particles in cell culture medium and/or intracellular compartments. Metal ions are known to interact with proteins to form aggregates, such as β-amyloid^31^. In the case of InCl_3_-exposed cells, the possibility that indium ions interact with proteins contained in FBS to form aggregates, which partially contribute to the genotoxicity, may not be neglected, but their contribution appears to be small under the conditions used. A549 cells have constitutively active Nrf-2^32^, which contributes to their protection against oxidative stress injury^33^. In this study, indium compounds caused clear nitrative DNA lesions in A549 cells, suggesting that indium-induced oxidative and nitrative stress overwhelmed their antioxidative potential.

In dose–response study, we demonstrated that indium compounds significantly increased 8-nitroG formation in A549 cells at an extremely low concentration of 5 ng/ml. We set 200 ng/ml as an optimal concentration for mechanistic studies, because all indium compounds more clearly induced 8-nitroG formation at this dose. A previous study has demonstrated indium-induced genotoxicity evaluated by Comet assay that detects strand breaks and alkali-labile sites at higher concentrations (200–400 µg/ml)^22,30^. These findings suggest that indium compounds induce different types of genotoxicity depending on their concentrations. Our conditions of 8-nitroG formation induced by indium compounds (5 ng/ml) are considered to be occupationally relevant according to the following estimation as shown in Table 1. According to recent study on personal indium exposure level in ITO workers (highest level: 24.0 µg/m^3^)^34^ and a particle deposition model^35^, we estimated that the level of In_2_O_3_ and ITO deposition in human alveoli can reach 1.42 ng/cm^2^ (equivalent to 5 ng/ml) in 0.225 and 0.195 years, respectively. This estimation was made by assuming that indium particles are evenly distributed on the alveolar surface and the clearance of these particles does not occur.

**Table 1:** Estimation of alveolar In_2_O_3_ and ITO deposition in exposed individuals.

Factors			References
(A) Maximum airborne indium concentration	24.0 µg/m^3^		Iwasawa et al.^34^
(B) Minute ventilation	0.02 m^3^/min		
	In_2_O_3_	ITO	
(C) Diameter of agglomerates	214.5 nm	194.5 nm	In this study
(D) Alveolar deposition efficiency	9%	10%	ICRP^35^
(E) Alveolar surface area	100 m^2^	100 m^2^	
(F) Alveolar deposition	0.0432 µg/min	0.0480 µg/min	(A) × (B) × (D)
	= 20.7 µg/day	= 23.0 µg/day	8 h/day
	= 104 µg/week	= 115 µg/week	5 days/week
	= 5.20 mg/year	= 5.75 mg/year	50 weeks/year
	= 5.20 ng/cm^2^/year	= 5.75 ng/cm^2^/year	Per alveolar surface area (E)
(G) Dose at which 8-nitroG formation was increased (5 ng/ml)	1.42 ng/cm^2^	1.42 ng/cm^2^	See below*
(H) Indium contained in (G)	1.17 ng/cm^2^	1.12 ng/cm^2^	See below**
(I) Indium deposition reaches (G) in	**0.225 years**	**0.195 years**	(H)/(F)

The calculation was performed on the assumption that particles are evenly distributed in the alveoli, and actually, particles may be accumulated in particular sites and the concentration will exceed the above value in a shorter duration.

*(G) 8-NitroG formation was significantly increased at 5 ng/ml = 1 ng/0.2 ml/well (culture slide) = 1 ng/0.7 cm^2^ = **1.42 ng/cm**^**2**^.

**(H) 1.42 ng/cm^2^ of In_2_O_3_ and ITO particles contain: (Molecular weights of In_2_O_3_: 278; In: 115; O: 16.0) In_2_O_3_: 1.42 × 230/278 = **1.17 ng/cm**^**2**^ of indium. ITO: 1.42 × 230/278 × 0.95 = **1.12 ng/cm**^**2**^ of indium.

Indium-induced 8-nitroG formation was inhibited by 1400 W and Bay, suggesting that iNOS expression was essential for DNA damage. NF-κB regulates expression of various genes involved in inflammatory responses, including iNOS ^36,37^. Indium-induced 8-nitroG formation was also suppressed by MBCD, MDC and CytoD, suggesting that caveolae- and clathrin-mediated endocytosis was involved in DNA damage. Nanoparticles up to approximately 500 nm and 200 nm are primarily internalized by caveolae- and calthrin-mediated endocytosis respectively^38,39^. Regarding size distribution of indium compounds, the peaks for In_2_O_3_ and ITO were 214.5 and 194.5 nm, and Z-averages for In_2_O_3_ and ITO were 208.0 and 149.0 nm, respectively. Therefore, they are likely to be internalized via these types of endocytosis and cause DNA damage. We have previously reported that these endocytosis inhibitors largely reduced cellular uptake of nanoparticles into cultured cells as demonstrated by light and electron microscopy and flow cytometry, resulting in the reduction in 8-nitroG formation^27–29^. Therefore, 8-nitroG formation induced by indium compounds appears to be largely accounted for by cellular uptake of their particles.

We have previously reported that 8-nitroG was formed at the sites of inflammation-related carcinogenesis in various animal model and clinical specimens^20,40,41^. Our studies using cultured cells have shown that particulate materials, including MWCNT^27,28^, carbon black^29^ and In_2_O_3_^21^, induced 8-nitroG formation. Because the glycosidic bond between 8-nitroG and deoxyribose is chemically unstable, 8-nitroG can be spontaneously released from DNA, resulting in the formation of apurinic site^42^. During DNA synthesis, adenine is preferentially misincorporated opposite an apurinic site^43^ and intact 8-nitroG^44^, leading to G → T transversion. Thus, 8-nitroG is a potentially mutagenic DNA lesion and may contribute to indium-induced carcinogenesis.

The molecular mechanisms of indium-induced inflammatory responses have been investigated. Badding et al. have demonstrated that ITO induced pro-inflammatory responses via NLRP3 inflammasome activation in RAW 264.7 mouse macrophages and BEAS-2B human bronchial epithelial cells^45^. In this study, we focused on the role of the HMGB1-RAGE-TLR9 pathway in indium-induced DNA damage, as demonstrated in DNA damage in human lung epithelial cells treated with MWCNT^27^. HMGB1 is a nuclear protein released from damaged or necrotic cells and interacts with DNA to form the HMGB1-DNA complex^26^. In this study, MTT assay revealed that even high concentrations (up to 50 µg/ml) of indium compounds did not show significant cytotoxicity. Therefore, it is speculated that HMGB1 and DNA were released from damaged cells that are not deadly. RAGE is a multi-ligand transmembrane receptor on the cell membrane and constitutively expressed in the lung throughout the life^24^. TLR9 resides in endosomes and lysosomes and activates inflammatory responses via interaction with CpG DNA of exogenous and endogenous origin^24,25^. The HMGB1-DNA complex binds to RAGE and then activates TLR9-mediated inflammatory responses. In this study, indium-induced 8-nitroG formation in A549 cells was largely reduced by the transfection with siRNA for *HMGB1*, *AGER* and *TLR9* and the pretreatment with antibodies against HMGB1 and RAGE. This finding indicates that the HMGB1-RAGE-TLR9 signaling pathway plays a key role in indium-induced DNA damage. The transfection with negative control siRNA and treatment with isotype control IgGs did not affect 8-nitroG formation, confirming the involvement of this pathway in DNA damage.

**Figure 7:**
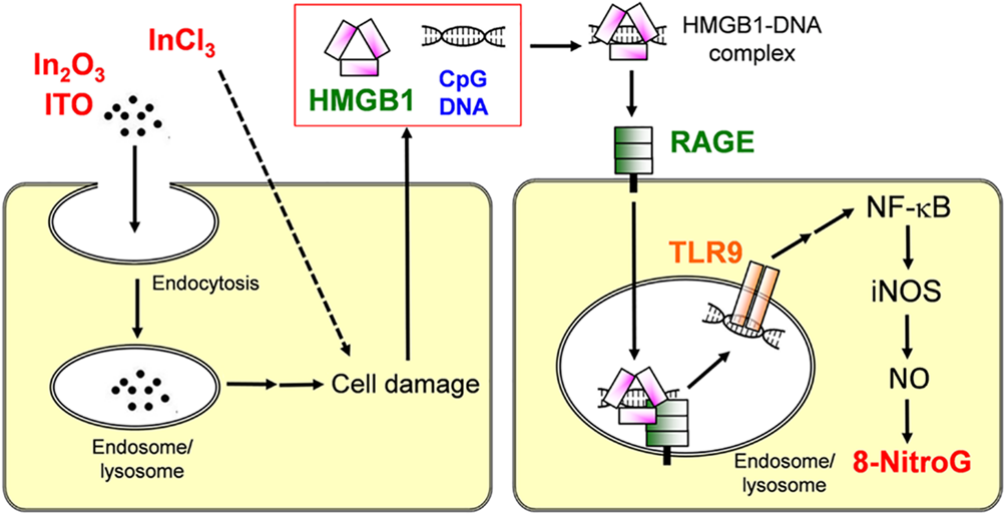
Proposed mechanism of indium-induced DNA damage in A549 cells.

Figure 7 shows the proposed mechanism of indium-induced DNA damage. In_2_O_3_ and ITO particles are taken up by the cell via endocytosis and InCl_3_ may enter the cell via diffusion, leading to cell injury. Cell injury caused by indium compounds may be accounted for by both their particulate properties and indium ions, derived from not only InCl_3_ but also the release from In_2_O_3_ and ITO particles. The HMGB1-DNA complex released from damaged cells is captured by RAGE on the surface of neighboring cells. This receptor is internalized into endosome and/or lysosome, where CpG is recognized by TLR9. TLR9 mediates NF-κB activation and iNOS expression, resulting in nitrative DNA damage. This molecular mechanism may contribute to indium-induced carcinogenesis. Endocytosis inhibitors reduced 8-nitroG formation in cells treated with indium compounds. This result may be explained by the inhibition of cellular uptake of the HMGB1-DNA complex into the cells.

## Conclusion

In this study, we first demonstrated that indium compounds induced 8-nitroG formation in human lung epithelial cells. It is noteworthy that both particles of indium compounds (In_2_O_3_ and ITO) and InCl_3_ caused clear 8-nitroG formation at extremely low doses regardless their chemical and physical properties. In addition, we found that the HMGB1-RAGE-TLR9 pathway plays a key role in indium-induced DNA damage. These finding would provide an insight into the molecular mechanism of genotoxicity induced by a wide variety of industrial chemicals.

## Methods

### Preparation of indium particles

In_2_O_3_ and ITO nanoparticles were obtained from Nanostructured and Amorphous Materials, Inc. (purity > 99.99%, primary diameter: 30–50 nm, Houston, TX, USA). ITO nanoparticles contained 95% of In_2_O_3_ and 5% of SnO_2_. InCl_3_ was obtained from Kanto chemical Inc. (purity > 99.95%, Tokyo, Japan). In_2_O_3_ and ITO nanoparticles were suspended in DMEM (Gibco/BRL, New York, NY, USA) containing 5% (v/v) heat-inactivated FBS and 100 mg/l kanamycin as described previously^21^. The suspension was vortexed for 1 min and then sonicated for 20 min at 40 W with a cup horn sonicator (Advanced Sonifier Model 450 Branson Ultrasonic, Danbury CT, USA). The suspensions containing dispersed In_2_O_3_ and ITO particles and InCl_3_ solution were stored at − 80 °C until use. We thawed and vortexed these samples immediately before the experiments. Size distribution for agglomerates of In_2_O_3_ and ITO was measured with a Zetasizer Nano particle size analyzer (Malvern, Worcestershire, UK) under the same conditions as those used in experiments as described previously^21,27^.

### MTT assay

To evaluate cytotoxic effects of indium compounds, MTT assay was performed as reported previously^27^. A549 cells (1 × 10^4^ cells/well, RIKEN BioResource Center, Tsukuba, Japan) were cultured in DMEM containing 5% (v/v) FBS and 100 mg/l kanamycin in a 96-well plate overnight and treated with 5–50 µg/ml of indium compounds (In_2_O_3_, ITO and InCl_3_) for 24 h at 37 °C. The culture supernatant was removed and the cells were incubated with 0.5 mg/ml MTT for 4 h at 37 °C, followed by treatment with dimethylsulfoxide for 10 min at room temperature. The absorbance of each well was measured at 570 nm with a Model 680 microplate reader (Bio-Rad Laboratories, Hercules, CA, USA).

### Detection of 8-nitroG formation in A549 cells

To investigate the mechanism of indium-induced carcinogenesis, we performed an immunocytochemical analysis to detect the formation of 8-nitroG in A549 cells. A549 cells (0.2 × 10^6^ cells/ml) were cultured in DMEM containing 5% (v/v) FBS and 100 mg/l kanamycin in 8-well culture slides (BD Falcon, Franklin Lakes, NJ, USA) and incubated overnight at 37 °C. Then cells were treated with the indicated doses (5–200 ng/ml) of In_2_O_3_, ITO and InCl_3_ for indicated durations (2–8 h). Positive control for 8-nitroG formation was prepared by incubating A549 cells for 2 h at 37 °C with culture supernatant of MWCNT-exposed cells prepared as reported previously^27^. The culture supernatant was obtained after A549 cells were treated with 1 μg/ml of MWCNT for 8 h, and centrifuged to remove MWCNT. Then, the supernatant was used for the experiment.

To examine the effects of inhibitors of iNOS and endocytosis on indium-induced 8-nitroG formation, cells were co-treated with 1 µM 1400 W (an inhibitor of iNOS), 10 µM Bay (an inhibitor of NF-κB), 2 mM MBCD (an inhibitor of caveolae-mediated endocytosis), 50 µM MDC (an inhibitor of clathrin-mediated endocytosis) or 1 µM CytoD (an inhibitor of actin polymerization). We used these concentrations of inhibitors, because they did not show any significant cytotoxic effects^27^. These inhibitors were purchased from Sigma-Aldrich (St, Louis, MO, USA). After incubation, we dried the culture slides at 37 °C and treated with 4% (v/v) formaldehyde for 10 min. The cells were treated with 0.5% (v/v) Triton-X100 in phosphate-buffered saline (PBS, pH 7.4) for 3 min and then treated with 2 N HCl for 30 min to denature DNA so that the antibody can easily detect DNA lesions as described previously^46^. Then the cells were incubated with 1% (w/v) skim milk dissolved in PBS for 1 h at room temperature. We treated the cells with rabbit polyclonal anti-8-nitroG antibody (1 µg/ml) produced by our group^47,48^ overnight, followed by the incubation with fluorescent Alexa 594-anti-rabbit IgG antibody (1:400, Molecular Probes, Eugene, OR, USA) for 3 h. To stain the nucleus, cells were treated with 5 µM Hoechst 33258. The stained cells were examined under a fluorescence microscope (BX53, Olympus, Tokyo, Japan) with the exposure time of 600 and 40 ms for red and blue fluorescence, respectively. We employed these conditions because the difference and linearity in fluorescence intensity between control and indium-exposed samples were clearly observed. Staining intensity of 8-nitroG was quantified by analyzing 5 randomly selected fields per sample with an image J software as follows. The total fluorescence intensity of 8-nitroG in the image was quantified and the intensity of the background, where no cells exist, was subtracted. Then the image of the cell nuclei, stained with Hoechst 33258, was converted to a binary image and the area of the nuclei was quantified. Finally, the fluorescence intensity of 8-nitroG was divided by the area of nuclei.

### Inhibition of 8-nitroG formation in A549 cells by siRNA transfection

To investigate the involvement of HMGB1, RAGE and TLR9 in indium-induced 8-nitroG formation, A549 cells were transfected with 10 nM Silencer Select siRNA (Ambion, Austin, TX, USA) for *HMGB1* (s6645), *AGER* (for RAGE, s1166) and *TLR9* (s28873) by using Lipofectamine 3000 reagent (Invitrogen, Carlsbad, CA, USA) in Opti-MEM I medium (Gibco), and incubated for 2 days at 37 °C. To confirm the specificity of siRNA, the cells were transfected with 10 nM Negative control #2 siRNA (Ambion). Then we incubated the cells with 200 ng/ml of In_2_O_3_, ITO and InCl_3_ for 4 h. Then 8-nitroG formation was examined by immunocytochemistry as described above.

To confirm the inhibitory effects of siRNA on gene expression, we performed Western blotting as described previously^27,29^. A549 cells transfected with siRNA were lysed in RIPA buffer (Cell Signaling Technology, Danvers, MA, USA) and centrifuged at 14,000*g* for 10 min. The protein concentration in the supernatant was measured with Coomasie Protein Assay Reagent Kit (Pierce Biotechnology, Rockford, IL, USA). Proteins were separated by 5–20% SDS-PAGE (SuperSep Ace, Wako Pure Chemical Industries, Osaka, Japan), and blotted onto a polyvinylidene difluoride membrane. The membrane was treated with 5% (w/v) skim milk in Tris-buffered saline (pH 7.4) containing 0.1% (v/v) Tween 20. Then the membrane was incubated with anti-HMGB1 mouse monoclonal antibody (1:500, ab77302, Abcam, Cambridge, UK), anti-RAGE mouse monoclonal antibody (1:500, ab54741, Abcam) or anti-TLR9 rabbit polyclonal antibody (1:500, ab37154, Abcam) along with anti-GAPDH rabbit polyclonal antibody (1:1,000, Santa Cruz Biotechnology, Santa Cruz, CA, USA) for 1 h. The membrane was then treated with horseradish peroxidase-conjugated anti-rabbit IgG antibody (1:10,000; Santa Cruz Biotechnology) and/or anti-mouse IgG antibody (1:2,000, Santa Cruz Biotechnology) for 30 min. Finally, we treated the membrane with ECL Western Blotting Detection Reagents (GE Healthcare, Buckinghamshire, UK) and analyzed with a LAS-4000 mini imager (Fujifilm, Tokyo, Japan). We measured the band intensity with an image J software and normalized with GAPDH.

### Blocking of 8-nitroG formation in A549 cells using anti-HMGB1 and RAGE antibodies

To examine the role of HMGB1 and RAGE in 8-nitroG formation, A549 cells were pretreated with 10 μg/ml anti-HMGB1 (ab77302, Abcam Cambridge, UK) and 10 μg/ml anti-RAGE (ab54741, Abcam, Cambridge, UK) antibodies. We also used the corresponding isotype control IgGs [mouse IgG_1_ (ab18447, Abcam) for anti-HMGB1 antibody and IgG_2a_ (ab18414, Abcam) for anti-RAGE antibody] to confirm the specificity of these antibodies. Then the cells were incubated with 200 ng/ml of In_2_O_3_, ITO and InCl_3_ for 4 h. Then 8-nitroG formation was examined by immunocytochemistry as described above.

### Statistical analysis

Statistical analysis was performed by analysis of variance (ANOVA) followed by Tukey’s multiple comparison test using an SPSS software (20.0 for Mac) as described previously^27^. Results were presented as means ± SD. *P* values less than 0.05 were considered to be statistically significant.

## Supplementary information

Supplementary Figure S1

Supplementary Figure S2

## Data Availability

Correspondence and requests for further data should be available address to Y.H.
