# Supplementary figures and images for: Nitrative DNA damage in lung epithelial cells exposed to indium nanoparticles and indium ions
Source: Sci Rep. 2020 Jul 1;10:10741. doi: 10.1038/s41598-020-67488-3 (PMC7329867; doi:10.1038/s41598-020-67488-3)

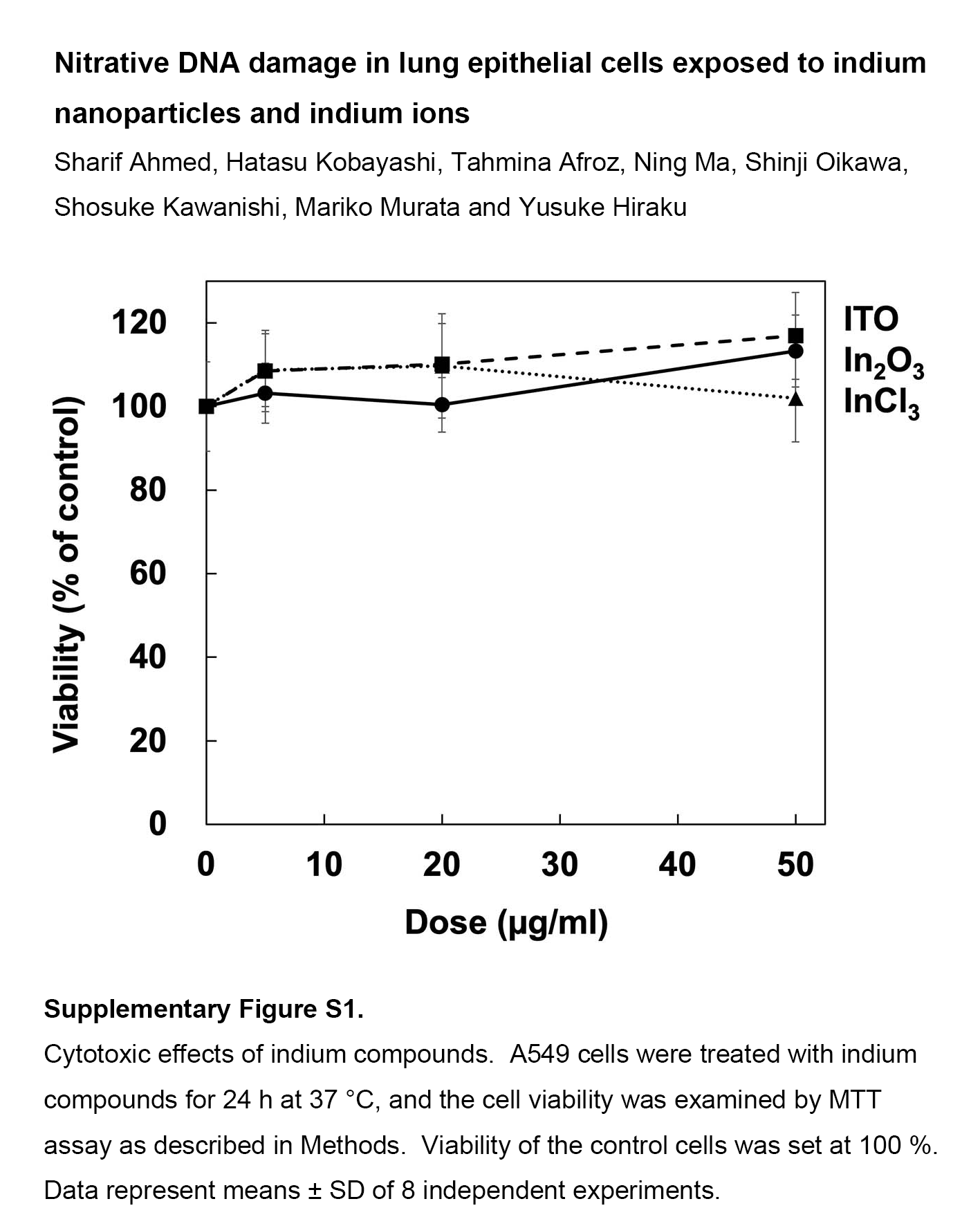

Supplement: Supplementary file 1 — Supplementary Figure S1 [file 41598_2020_67488_MOESM1_ESM.tif]

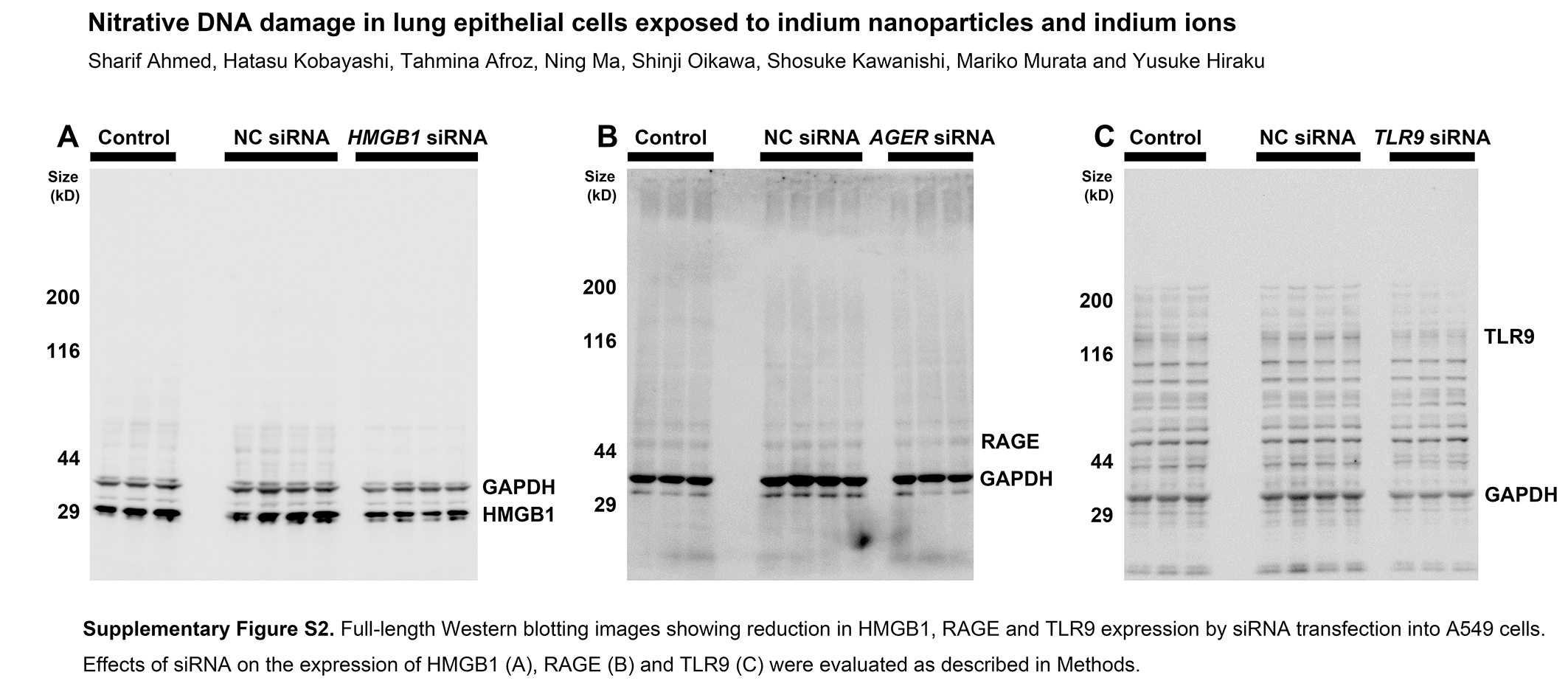

Supplement: Supplementary file 2 — Supplementary Figure S2 [file 41598_2020_67488_MOESM2_ESM.tif]
